# Case Report: Diagnostic pitfalls in localized aortic root dissection: two cases and a literature review

**DOI:** 10.3389/fcvm.2026.1769867

**Published:** 2026-03-24

**Authors:** Qiongwei Jia, Yizhong Bian, Xidan Wang, Tianhang Lan, Xianshuai Li, Lei Xu, Xiaoying Tao

**Affiliations:** 1 Department of Ultrasound Medicine, Affiliated Jinhua Hospital Zhejiang University School of Medicine, Jinhua, Zhejiang, China; 2 Zhejiang Chinese Medical University, Hangzhou, Zhejiang, China; 3 Department of Medical Imaging, Affiliated Jinhua Hospital Zhejiang University School of Medicine, Jinhua, Zhejiang, China; 4 Department of Cardiothoracic Surgery, Affiliated Jinhua Hospital Zhejiang University School of Medicine, Jinhua, Zhejiang, China

**Keywords:** aortic dissection, aortic valve regurgitation, case report, localized aortic root dissection, transesophageal echocardiography

## Abstract

**Background:**

Localized aortic root dissection is a rare and distinct variant of aortic dissection, characterized by an intimal tear confined to the aortic root without distal propagation. Its nonspecific presentation and subtle imaging findings frequently result in diagnostic error. This report describes two cases and reviews the literature to highlight key imaging pitfalls and diagnostic considerations.

**Case presentation:**

In the first patient, transthoracic echocardiography (TTE) and transesophageal echocardiography (TEE) initially misidentified the dissection as a congenital supravalvular aortic membrane, while coronary computed tomography angiography (CTA) did not identify a definite aortic root dissection. The correct diagnosis was established intraoperatively. In the second patient, TTE and CTA were non-diagnostic, whereas TEE subsequently visualized the localized dissection. Notably, neither patient had significant aortic root dilatation, classic “tearing” chest pain, or features of connective tissue disease.

**Conclusion:**

Localized aortic root dissection presents with atypical symptoms and subtle, potentially confusing imaging features, leading to misdiagnosis or delayed recognition. In patients with severe aortic regurgitation of unclear etiology, this entity should be considered even in the absence of aortic root dilatation or typical tearing chest pain. Comprehensive morphological assessment of the aortic root is crucial. TEE offers a higher diagnostic yield than TTE or CTA and is useful for detection and differentiation; when clinically stable, early comprehensive TEE should be considered.

## 1 Introduction

Aortic dissection, a major entity within the acute aortic syndrome (AAS) spectrum, is the most frequently encountered presentation. The estimated annual incidence is approximately 5–30 cases per million population. It most commonly affects individuals aged 50–70 years and shows a male predominance (1). Pathophysiologically, an intimal tear permits blood to enter the medial layer, creating a false lumen; the dissection flap may extend antegrade or retrograde and can result in life-threatening complications. Established risk factors include hypertension, atherosclerosis, and inherited connective tissue disorders, the majority of patients present with sudden, severe chest and back pain, often described as “tearing” in nature, and frequently have a history of arterial hypertension (2).

Localized aortic root dissection is an exceedingly rare and distinct variant of aortic dissection, characterized by an intimal tear confined to the aortic root, usually just above the aortic annulus, without distal propagation. Clinical manifestations are often atypical, which increases the risk of missed or incorrect diagnosis. The available evidence is limited to case reports, and no systematic epidemiological studies have been published. Here, we describe the imaging features and clinical management of two such cases. By synthesizing the existing literature, we aim to provide echocardiography-focused diagnostic guidance for this variant and thereby improve diagnostic accuracy.

## 2 Case description

### 2.1 Case 1

A 36-year-old man presented with a 2-month history of recurrent chest tightness and dyspnea, with recent worsening. He was initially diagnosed with a pulmonary infection. He had no relevant medical history, denied any prior episodes of abrupt chest pain, and was a healthy non-smoker.

#### 2.1.1 Clinical examination and initial laboratory findings

On physical examination, the patient was tachycardic and had a diastolic decrescendo murmur best heard at the lower left sternal border. Twenty-four-hour ambulatory blood pressure monitoring showed a mean daytime blood pressure of 128/71 mmHg. Laboratory testing revealed mildly elevated cardiac enzyme levels, whereas the D-dimer level was within the normal range on admission. The electrocardiogram showed sinus rhythm with U-wave changes and QT prolongation.

#### 2.1.2 Transthoracic and transesophageal echocardiography

**Figure 1:**
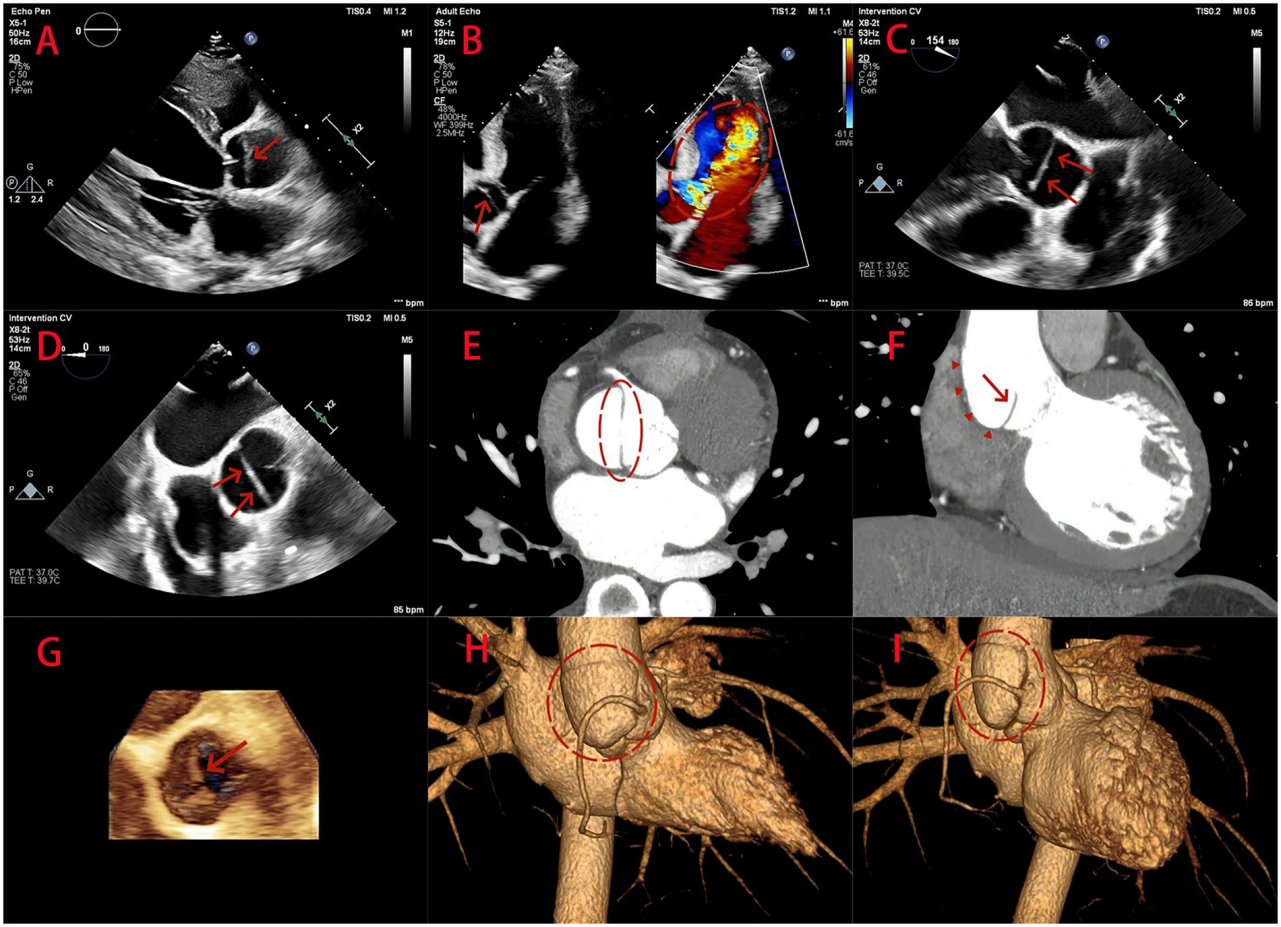
Echocardiographic and coronary CTA findings in a 36-year-old man with localized aortic root dissection. **(A)** Admission transthoracic echocardiography (TTE) shows a membrane-like structure (red arrow) at the sinotubular junction of a non-dilated aortic root, oriented perpendicular to the long axis of the ascending aorta. **(B)** Apical five-chamber view demonstrates the membrane-like structure (red arrow) more clearly, with significant regurgitation originating from both the membrane and the aortic valve (red circle). **(C,D,G)** Preoperative transesophageal echocardiography (TEE) in two- and three-dimensional views delineates the membrane-like structure (red arrow), extending toward the left–noncoronary commissure and into the right coronary sinus. **(E,F)** Retrospective review of the preoperative coronary CTA shows a localized, obliquely oriented intimal flap at the aortic root (red arrow) on coronal and axial images, associated with focal outward bulging of the aortic wall (red triangle). **(H,I)** Three-dimensional reconstructions demonstrate focal outward bulging of the adjacent aortic root wall in the dissected segment (red circle).

Transthoracic echocardiography (TTE) on admission showed an aortic root diameter of 37 mm without overt dilatation. A thin, membrane-like structure was visualized at the sinotubular junction, oriented perpendicular to the long axis of the ascending aorta (Figure 1A). Significant aortic regurgitation originating from both the membrane-like structure and the aortic valve itself was observed (Figure 1B). Doppler assessment of transvalvular flow showed a peak velocity of 1.6 m/s, which was within the normal range and did not support hemodynamically significant aortic stenosis. Preoperative transesophageal echocardiography (TEE) clearly demonstrated the membranous structure in both two- and three-dimensional short-axis views of the aortic root. The structure was seen extending to the left-noncoronary commissure and the right coronary sinus (Figures 1C–G).

TTE also showed left ventricular dilatation, whereas the left ventricular ejection fraction remained within the normal range. Based on the overall clinical and echocardiographic findings, the working diagnosis was a congenital heart disease involving a supravalvular aortic membrane complicated by severe aortic regurgitation.

#### 2.1.3 Preoperative computed tomography angiography (CTA), intraoperative findings, and retrospective CTA review

Following the abnormal findings on TTE and further evaluation by TEE, preoperative coronary CTA was performed to evaluate the presence of concomitant coronary artery disease and to facilitate perioperative risk stratification and surgical planning. The initial CTA report showed no significant coronary stenosis but noted cardiomegaly and a small pericardial effusion. No definite aortic dissection was recognized preoperatively.

**Figure 2:**
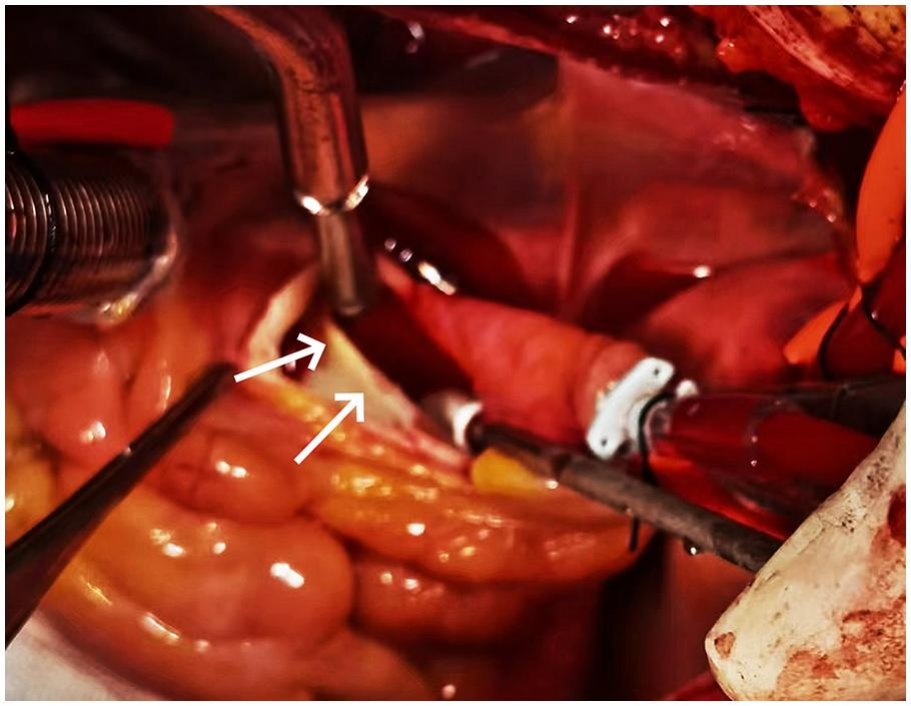
Intraoperative photograph of Case 1 showing an intimal flap confined to the aortic root (white arrow).

Despite the absence of a definitive dissection diagnosis on preoperative CTA, surgery was indicated because of severe aortic regurgitation with progressively worsening chest tightness and dyspnea, in conjunction with cardiomegaly, a pericardial effusion, and a structurally abnormal membrane-like lesion at the aortic root on echocardiography. Intraoperatively, after opening the aortic root, a highly localized aortic root dissection was identified without evidence of an intramural hematoma. The membrane-like structure initially suspected on echocardiography to represent a congenital supravalvular aortic membrane was confirmed to be the intimal flap (Figure 2). The intimal entry tear was located near the sinotubular junction, and the flap extended retrogradely toward the right coronary ostium and the commissure between the left and right coronary cusps. Mild prolapse of these cusps was also noted, likely reflecting localized annular instability and contributing to severe aortic regurgitation.

The lesion was managed with repair of the aortic root entry tear using felt reinforcement, followed by mechanical aortic valve replacement, which eliminated aortic regurgitation. Postoperative histopathological examination of the excised valve tissue showed collagenization with mucoid degeneration (Figure 4A). The postoperative course was uneventful; the patient was discharged on postoperative day 8 and remained well at follow-up.

After the intraoperative diagnosis was established, the preoperative CTA dataset was retrospectively re-evaluated with a specific focus on the aortic root using multiplanar and three-dimensional reconstructions. This targeted review revealed a localized, obliquely oriented intimal flap at the aortic root on both coronal and axial images (Figures 1E,F), together with focal outward bulging of the adjacent aortic wall (Figures 1H,I). Failure to recognize the lesion on the initial CTA interpretation was likely attributable to its extremely limited extent and subtle imaging features, which became appreciable only after dedicated aortic root reconstruction and focused re-assessment.

### 2.2 Case 2

A 68-year-old man presented with a 2-day history of chest tightness and dyspnea. He was initially diagnosed with heart failure. He denied any abrupt-onset chest pain. His medical history was notable for untreated hypertension, and the level of blood pressure control was unknown. No other relevant medical or family history was reported.

#### 2.2.1 Clinical examination and initial laboratory findings

On physical examination, a holodiastolic decrescendo murmur was heard best at the left sternal border in the third and fourth intercostal spaces, with radiation to the apex. Coarse crackles were present in both lung fields. On admission, the blood pressure was 109/61 mmHg. Laboratory testing showed mildly elevated cardiac enzyme levels and an elevated D-dimer level. The electrocardiogram showed no significant ST-segment or T-wave abnormalities.

#### 2.2.2 Transthoracic and transesophageal echocardiography

**Figure 3:**
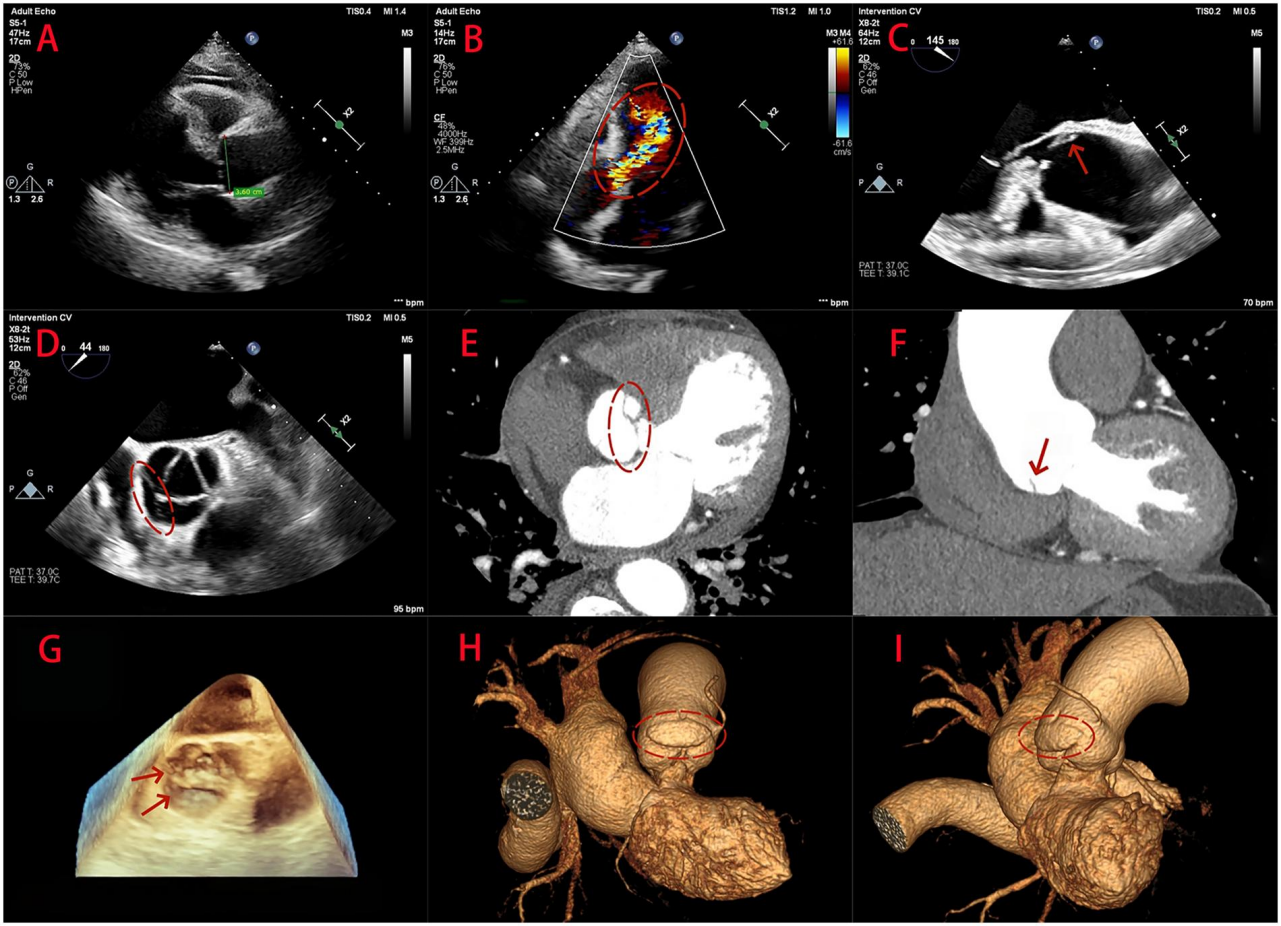
Echocardiographic and coronary computed tomography angiography (CTA) findings in a 68-year-old man with localized aortic root dissection. **(A)** Emergency transthoracic echocardiography (TTE) on admission shows no aortic root dilatation, with the sinuses of Valsalva measuring 36 mm. **(B)** Doppler demonstrates severe aortic regurgitation (red circle). **(C,D,G)** Preoperative transesophageal echocardiography (TEE) in two- and three-dimensional views demonstrates a distinct, obliquely oriented membranous structure confined to the aortic sinus, involving the right and non-coronary sinuses (red arrows/circle). **(E,F)** Retrospective review of the preoperative coronary CTA identifies a localized, obliquely oriented intimal flap within the aortic sinuses on coronal and axial images (red arrow). **(H,I)** Three-dimensional reconstructions demonstrate mild focal bulging of the involved sinus walls in the dissected segment (red circle).

Emergency TTE on admission showed no aortic root dilatation, with the sinuses of Valsalva measuring 36 mm (Figure 3A). While the aortic valve structures were suboptimally visualized, the examination indicated findings suggestive of mild aortic stenosis and severe aortic regurgitation (Figure 3B). Subsequent preoperative TEE provided better delineation on both two- and three-dimensional views and demonstrated a distinct, obliquely oriented membranous structure confined to the aortic sinus, involving the right and non-coronary sinuses (Figures 3C–G). The commissural region between the right and non-coronary cusps were fused to this membranous structure, resulting in severe aortic regurgitation. Based on the overall clinical and echocardiographic assessment, localized aortic sinus dissection extending into the valvular apparatus was considered, complicated by mild aortic valve stenosis and severe regurgitation.

#### 2.2.3 Preoperative CTA, intraoperative findings, and retrospective CTA review

Following the abnormal findings on TTE and subsequent TEE, preoperative coronary CTA was performed as part of the surgical work-up. The scan showed no significant coronary stenosis but noted cardiomegaly and a pericardial effusion. Three-dimensional reconstruction of the thoracoabdominal aorta was unremarkable, and no definite aortic dissection was recognized on the initial CTA assessment.

**Figure 4:**
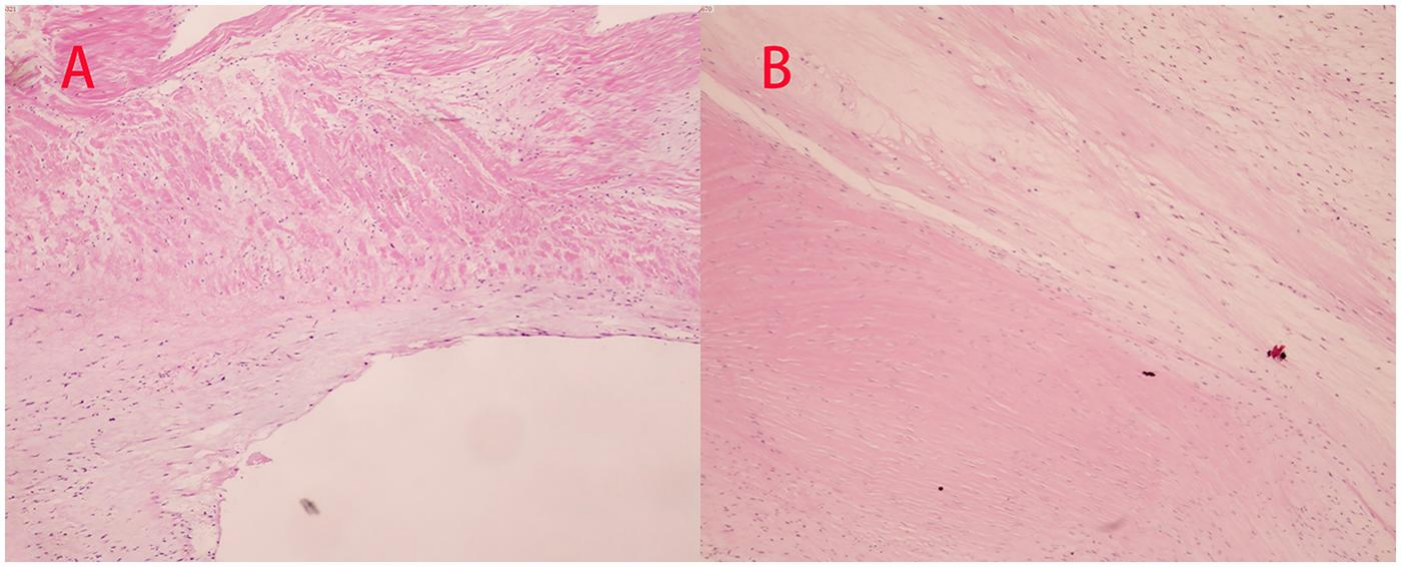
Postoperative histopathological findings of the two cases. **(A)** Hematoxylin and eosin (H&E) staining (×10) of the aortic valve tissue from Case 1 shows collagenization with mucoid degeneration. **(B)** H&E staining (×20) of the aortic valve tissue from Case 2 shows collagenization with focal calcification.

Based on the preoperative echocardiographic impression and the severity of aortic valve dysfunction, surgery proceeded as planned. Intraoperative inspection revealed a transverse intimal entry tear within the aortic sinus, involving the right coronary and non-coronary sinuses over an extent of approximately 2.5 cm. The aortic valve showed mild calcific stenosis with severe regurgitation. The lesion was managed with repair of the sinus tear using felt reinforcement, followed by mechanical aortic valve replacement, which eliminated the regurgitation. Histopathological examination of the excised valve tissue showed collagenization with focal calcification (Figure 4B). The postoperative course was uneventful; the patient was discharged on postoperative day 7 and has remained well on follow-up.

After the intraoperative diagnosis was established, the preoperative CTA dataset was retrospectively re-evaluated with a specific focus on the aortic root using multiplanar and three-dimensional reconstructions. This targeted review identified a localized, obliquely oriented intimal flap within the aortic sinuses on both coronal and axial images (Figures 3E,F), accompanied by mild focal bulging of the involved sinus walls (Figures 3H,I). The lesion was not recognized on the initial CTA interpretation, likely because of its extremely limited extent and subtle imaging features, which became appreciable only after dedicated aortic root reconstruction and focused re-assessment.

## 3 Discussion and summary

In clinical practice and research, aortic dissection is most commonly classified into two major systems: the Stanford and DeBakey classifications. Among these, aortic dissection confined exclusively to the ascending aorta (DeBakey type II) represents the rarest subtype, comprising only about 5.8% of all cases (3). In our two patients, the lesion was confined to the aortic root without extension beyond the sinotubular junction. Localized aortic root dissection therefore represents an exceptionally rare and highly restricted form of DeBakey type II dissection. The first documented case was reported in 1975 (4), establishing this entity in the literature. Subsequent case reports have been published, and a search of PubMed, Web of Science, and ProQuest databases up to October 2025 identified 33 cases with detailed diagnostic and management documentation. In our review of the literature, we focused on spontaneous localized aortic root dissection; iatrogenic cases related to catheter-based procedures were not included.

In contrast to most prior reports, neither of our patients presented with significant aortic root dilatation, typical tearing chest pain, or features of connective tissue disease. The second patient had a history of hypertension, which may have contributed to the development of this localized dissection. In both cases, the lesions were considered more likely to represent a chronic or subacute-to-chronic process rather than a typical acute dissection, based on the highly localized morphology, atypical clinical presentation, and intraoperative assessment by the operating surgeon. However, because the exact onset could not be determined retrospectively, this interpretation should be considered cautious. The true incidence remains unknown.

This localized dissection may result from an interplay between cystic medial degeneration and hypertension (5). However, the precise mechanisms preventing distal propagation from the intimal tear site remain unclear. Some authors have hypothesized that a larger, transversely oriented intimal tear may constrain the dissection circumferentially (6–10). As summarized in Table 1, initial misdiagnosis is common (81.8%), highlighting the need for a high index of suspicion and careful aortic root imaging assessment. Regarding aortic root size, dilatation was reported in 17 of 33 cases, and exact aortic root diameter values were available in 15 patients, with a median diameter of 50.0 mm (IQR: 45.5–52.0 mm; range: 45.0–80.0 mm). The other reported cases did not exhibit aortic root dilatation, and our two patients likewise belonged to this non-dilated subgroup. Notably, neither lesion was recognized on the initial interpretation of preoperative coronary CTA. Although our sample is limited, these observations support the possibility that a subset of localized aortic root dissections may follow a subacute-to-chronic trajectory, which may contribute to atypical presentations and delayed recognition; this inference remains cautious given uncertainty in symptom onset.

**Table 1:** Summary of the clinical and imaging characteristics of reported cases (*n* = 33).

Clinical and imaging characteristics	Number/Total (%)
Mean age (years)	53.8
Gender	
Male	25/33 (75.8%)
Female	8/33 (24.2%)
Relevant Medical History	
No relevant history reported	16/33 (48.5%)
Hypertension	15/33 (45.5%)
Bicuspid aortic valve	2/33 (6.1%)
Takotsubo cardiomyopathy	1/33 (3.0%)
Eosinophilic granulomatosis with polyangiitis	1/33 (3.0%)
Autosomal dominant polycystic kidney disease	1/33 (3.0%)
Symptom	
Chest pain	14/33 (42.4%)
Dyspnea	8/33 (24.2%)
Chest tightness	5/33 (15.1%)
Asymptomatic	2/33 (6.1%)
Back pain	1/33 (3.0%)
Dizziness and diaphoresis	1/33 (3.0%)
Cardiopulmonary arrest	1/33 (3.0%)
Jaw pain	1/33 (3.0%)
Epigastric pain	1/33 (3.0%)
Misdiagnosis at first visit	27/33 (81.8%)
Initial working diagnosis	
Severe aortic regurgitation of unknown etiology	16/33 (48.5%)
Acute coronary syndrome	8/33 (24.2%)
Aortic dissection	6/33 (18.2%)
Endocarditis	2/33 (6.1%)
Pericarditis	1/33 (3.0%)
Congenital supravalvular aortic membrane	1/33 (3.0%)
Ascending aortic aneurysm	1/33 (3.0%)
Aortic valve prolapse	1/33 (3.0%)
Cardiac tamponade	1/33 (3.0%)
Echocardiography findings	
Severe aortic valve regurgitation	24/33 (72.7%)
Intimal flap	23/33 (69.7%)
Aortic root dilatation	17/33 (51.5%)
Pericardial effusion	2/33 (6.1%)
Aortic root hematoma	2/33 (6.1%)
CT findings	
Intimal flap	20/33 (60.6%)
Aortic root dilatation	12/33 (36.4%)
Localized bulging of the aortic root wall	2/33 (6.1%)
Diagnostic modality	
TEE	13/33 (39.4%)
TTE	6/33 (18.2%)
CTA	6/33 (18.2%)
Intraoperative	5/33 (15.2%)
Aortic root angiography	2/33 (6.1%)
Intravascular ultrasound	1/33 (3.0%)
Treatment	
Surgery	31/33 (93.9%)
Conservative management	1/33 (3.0%)
No intervention	1/33 (3.0%)

Consistent with this, accurate characterization of aortic root pathology remains challenging, largely due to the anatomical complexity of the region and the rarity of this condition. Localized aortic root dissection is therefore frequently overlooked or misinterpreted, and patients may be diagnosed instead with severe aortic regurgitation of unknown etiology or acute coronary syndrome. Even when an intimal flap is visualized, the unusual presentation may still lead to misinterpretation as alternative pathologies such as a congenital supravalvular aortic membrane (CSAM) (11), infective endocarditis (7), or pericarditis (12). There are also reported cases in which aortic valve chordae tendineae (13) or aortic valve commissural tears (14) have been mistaken for localized aortic root dissection.

In the first case we reported, the localized aortic root dissection was initially mistaken for a CSAM. However, congenital aortic membranes are typically located below the aortic valve and are more frequently associated with left ventricular outflow tract obstruction than with regurgitation (15). CSAM represents an exceptionally rare form of supravalvular aortic stenosis (SVAS) (16), characterized by luminal narrowing at the sinotubular junction leading to obstructive hemodynamics. In cases described in the literature (17, 18), the membrane has consistently been observed at the sinotubular junction and oriented perpendicular to the aortic long axis.

Morphologically, localized aortic root dissection must also be distinguished from aortic valve chordae tendineae and aortic valve commissural tears. Aortic valve chordae tendineae typically present as elongated, band-like structures extending from the aortic wall to the valve leaflets, with a predominant attachment to the non-coronary cusp (13). In contrast, the intimal flap of aortic root dissection appears as planar or lamellar structures with discernible thickness, frequently oriented obliquely or perpendicularly to the aortic wall. Notably, the observation of prolapse involving two aortic valve leaflets in the absence of an intimal tear should raise suspicion for commissural tear of the aortic valve (19).

In addition to the diagnostic challenges related to anatomy and hemodynamics, each imaging modality has technical limitations. Echocardiographic assessment of the aortic root is susceptible to artifact, and cyclic motion of the aortic root and ascending aorta may generate motion artifacts that obscure subtle intimal flaps. This issue is particularly relevant in localized aortic root dissection, because these lesions often do not show a classic intimal flap or double lumen in the distal ascending aorta. Consequently, such cases are prone to being missed on CTA or misinterpreted as motion artifacts or normal anatomy. The use of electrocardiogram (ECG)-gated CTA can substantially mitigate this diagnostic uncertainty (20).

Our literature review showed that TEE had a higher diagnostic yield for localized aortic root dissection than TTE or CTA (39.4%, 18.2%, and 18.2%, respectively). Accordingly, once clinically stable, patients with suspected localized aortic root dissection should undergo comprehensive TEE as early as possible.

From a management standpoint, surgery remains the predominant strategy for spontaneous localized aortic root dissection, given diagnostic uncertainty and the frequent presence of severe aortic regurgitation or other complications. To date, only one published spontaneous case has reported successful conservative management with favorable follow-up (21). A few iatrogenic aortic root dissections related to catheter-based procedures have also been managed non-operatively in stable patients (22). One hypothesis is that retrograde catheter-related injury creates an entry orientation that is unfavorable for antegrade flow to enter the tear, thereby favoring spontaneous sealing in selected uncomplicated cases (23). Nonetheless, evidence supporting conservative therapy remains limited to highly selected case reports and should be considered only in carefully chosen stable patients under strict hemodynamic control and close imaging surveillance.

From a surgical planning perspective, preoperative recognition of localized aortic root dissection may not change the indication for surgery when severe aortic valve dysfunction is already present, but it can improve diagnostic confidence and optimize perioperative preparation. In our two cases, both patients already had clear operative indications because of severe valvular dysfunction. However, earlier recognition of the localized dissection would have allowed more explicit preparation for possible aortic root or proximal aortic reconstruction. The final operative strategy still depends on the intraoperative extent of the lesion and the feasibility of durable repair.

Based on our cases and previous reports of diagnostic oversight, localized aortic root dissection should be included in the differential diagnosis in patients with severe aortic regurgitation of unclear etiology, even in the absence of aortic root dilatation or typical pain. A structured approach combining focused TEE assessment and targeted review of available coronary CTA datasets with dedicated aortic root reconstruction may improve diagnostic confidence, prioritizing detection of subtle intimal tears while accounting for imaging artifacts and anatomical variants that may mimic or obscure the lesion. Close preoperative communication between echocardiography and cross-sectional imaging teams is essential to minimize diagnostic oversight and optimize perioperative planning.

## 4 Conclusion

Localized aortic root dissection is a rare and easily overlooked subtype of aortic dissection that often presents with atypical clinical and imaging features. It may occur without typical tearing chest pain, and imaging findings can be subtle, requiring careful differentiation from CSAM, other mimics, and imaging artifacts. Our cases support the possibility that some localized aortic root dissections follow a chronic or subacute-to-chronic course, which may contribute to atypical presentations and delayed or incidental recognition. In patients with severe aortic regurgitation of unclear etiology, regardless of aortic root dilatation, clinicians should maintain a high index of suspicion and perform targeted aortic root assessment, particularly with comprehensive TEE. In future similar cases, earlier inclusion of this entity in the differential diagnosis and closer coordination between echocardiography and imaging review may improve diagnostic accuracy.

## Data Availability

The original contributions presented in the study are included in the article/Supplementary Material, further inquiries can be directed to the corresponding authors.
